# Substrate Effects on the Formation Process, Structure and Physicochemical Properties of Supported Lipid Bilayers

**DOI:** 10.3390/ma5122658

**Published:** 2012-12-07

**Authors:** Ryugo Tero

**Affiliations:** Electronics-Inspired Interdisciplinary Research Institute (EIIRIS), Toyohashi University of Technology, 1-1 Hibarigaoka, Tempaku-cho, Toyohashi 441-8580, Japan; E-Mail: tero@eiiris.tut.ac.jp; Tel.: 81-532-81-5136; Fax: 81-532-81-5141.

**Keywords:** lipid bilayer membranes, silicon oxide, mica, titanium dioxide, atomic force microscope, fluorescence microscope, single molecule tracking, surface hydrophilicity, anomalous diffusion, phase separation

## Abstract

Supported lipid bilayers are artificial lipid bilayer membranes existing at the interface between solid substrates and aqueous solution. Surface structures and properties of the solid substrates affect the formation process, fluidity, two-dimensional structure and chemical activity of supported lipid bilayers, through the 1–2 nm thick water layer between the substrate and bilayer membrane. Even on SiO_2_/Si and mica surfaces, which are flat and biologically inert, and most widely used as the substrates for the supported lipid bilayers, cause differences in the structure and properties of the supported membranes. In this review, I summarize several examples of the effects of substrate structures and properties on an atomic and nanometer scales on the solid-supported lipid bilayers, including our recent reports.

## 1. Introduction

Cell membranes are the reaction fields of the transportation of materials, signals and energy into and out of cells. The fundamental structure of cell membranes is the bimolecular sheet of amphiphilic lipid molecules, and various kinds of proteins are incorporated in and on the lipid bilayer membranes. The lateral and/or vertical structures and dynamics in the lipid bilayers, e.g., two-dimensional domain formation, hydrophobic matching, curvature stress, lateral diffusion of molecules and inter-leaflet flip-flop [1,2,3,4], are crucial factors during the reactions in cell membranes. Supported lipid bilayers (SLBs), which are artificial lipid bilayer membranes existing at the solid-liquid interfaces, are used to investigate the fundamental physicochemical properties of lipid bilayers and are also used as cell membrane model systems *in vitro*. Lipid molecules in SLBs do not directly adsorb on the solid substrate, but are separated from it by 1–2 nm thick water layer [5,6,7,8,9,10,11], therefore, the SLBs are available for the studies of the dynamics in lipid bilayer membranes, such as lipid diffusion and domain formation. For example, atomic force microscopy (AFM) is a typical surface scientific technique for the study of membrane morphology, domain structures and molecular distribution in SLBs [12,13,14]. The lipid diffusion and diffusion coefficients in SLBs are generally evaluated with fluorescence-based techniques, like fluorescence recovery after photobleaching [15,16], fluorescence correlation spectroscopy [17], and single molecule tracking [18]. In addition, surface patterning and machining techniques can be applied to the area-selective SLB formation and manipulation of molecules in SLBs [19,20,21,22,23,24,25,26,27,28,29,30,31,32]. These SLB devices are expected as a powerful tool for the analysis of membrane proteins, which account for roughly half of the targets of drug discovery [33].

It has been reported that, despite the existence of the water layer, the structures and properties of SLBs are affected by the physical and chemical properties of solid substrates. Recent studies showed unique substrate-induced phenomena of SLBs, which do not occur in the free-standing membranes; for example, decoupled phase transition [34,35,36,37] and asymmetric molecular distribution between the upper and lower leaflet of a SLB [38,39,40]. Understanding the interaction between solid substrates and lipid bilayers are important to realize artificial cell membrane systems on functional solid devices avoiding the denaturing of lipids and proteins and, furthermore, to control the bilayer membranes with functional substrates, because cell membranes are laterally heterogeneous and vertically asymmetric.

In this review, I summarize the effects of substrate structures and properties on the atomic and nanometer scales on the solid-supported lipid bilayers, including our recent reports. Even the surfaces of mica and a SiO_2_ layer on a Si wafer, which are flat, biologically inert and the most widely used substrates in SLB studies, show differences in the physical structure and properties of the supported membranes. First, we briefly describe the preparation of SLBs by the vesicle fusion method and how the SLB formation process is affected by solid substrates. Second, I describe the effects of substrates on the lateral diffusion of lipids and proteins in SLBs. Finally, the dependence of the two-dimensional domain formation in SLBs on substrate materials and their structures is presented. It also relates to the chemical reactivity of SLB to peptides.

## 2. Substrate Effects on SLB Formation Process

### 2.1. Vesicle Fusion Method for SLB Formation

**Figure 1:**
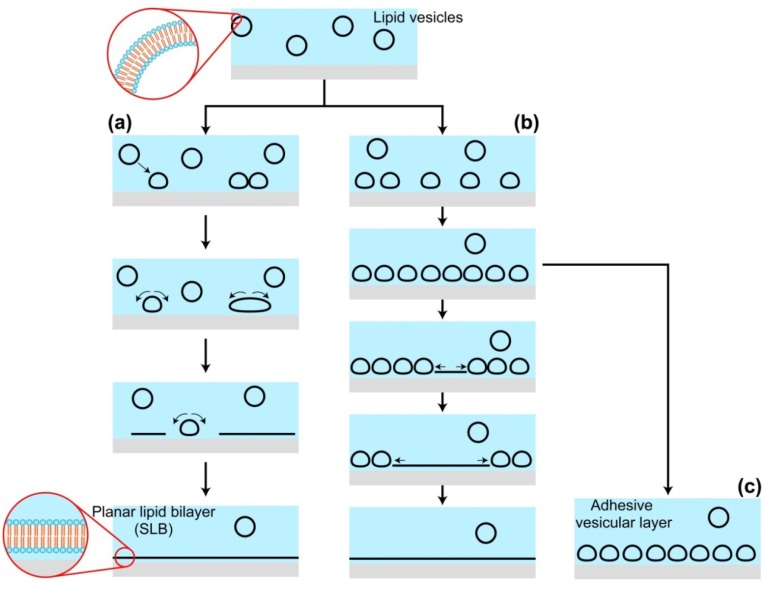
Schematic drawing of the shape transformation of lipid vesicles at a liquid-solid interface during the SLB formation by the vesicle fusion method. (**a**) Individual adsorbed vesicle ruptures and transforms to a SLB patch; (**b**) Vesicles adsorb until their coverage reaches to a threshold, and the rupture of a vesicle triggers the SLB growth by stimulating the neighboring vesicles; (**c**) A stable layer of adhesive vesicles, which does not transform to a planar lipid bilayer.

Lipid vesicles, also called liposomes, are spherical lipid bilayers dispersed in aqueous solutions. On a hydrophilic surface, vesicles fuse with each other and/or rupture, then transform to planar membrane under appropriate conditions (Figure 1). This spontaneous shape transformation from vesicles to planar membrane is applied to the formation of SLB on solid substrates and called the vesicle fusion method. Historically, to my knowledge, the first report of SLB formation by vesicle fusion is reference [41] by Brian and McConnell. (Neither lipid monolayer or bilayer forms are specified in this paper, but McConnell referred to it as the method for the bilayer formation in a later review [42].) Other methods for the SLB fabrication are the Langmuir-Schaefer/Blodgett technique [43,44], fusion of vesicles onto a lipid monolayer on a solid substrate [45] and the self-spreading from a bulk lipid source [46]. The advantages of the vesicle fusion method are: the formation of homogeneous SLB is possible independently of the size of substrates; SLBs are formed either flat surfaces or surfaces with three-dimensional structures; and, prepared lipid bilayers are free of organic solvent, thus the denaturing of proteins due to the organic solvents is avoided in case proteins are incorporated into lipid bilayers.

Lipid vesicles are prepared by the agitation of a dried film or a powder of lipids in aqueous solution. First, the solution of lipids in chloroform, ethanol or their mixture with the required amounts and components is taken and/or mixed in a glass vial, and the solvent is evaporated by N_2,_ followed by evacuation in vacuum. Water or a buffer solution is added to the glass vial, and the vial is agitated to suspend the lipids into the solution. The obtained turbid suspension contains multilamellar vesicles with various sizes, shapes and lamellar numbers. The suspension is treated with freeze-and-thaw cycles followed by extrusion through polycarbonate filters, or with sonication, to prepare unilamellar vesicles with a uniform size [47,48,49]. The extruded vesicles have similar diameters to the filter mesh size [48,49]. Sonication using a bath-type or tip-type sonicator reduces the vesicle size to less than 100 nm, depending on the output power of the sonicator [47,48]. The substrates for the SLB should be cleaned carefully to obtain contamination-free surfaces, with boiling in piranha solution (mixture of concentrated H_2_SO_4_ and H_2_O_2_ aqueous solution), UV-ozone ashing or plasma ashing, except for mica, which should be cleaved to expose fresh surface just before use. The substrates are incubated in the vesicle suspension at temperature above the phase transition temperature (*T*_c_) from gel phase to liquid crystalline phase (*L*_α_) of the lipid to keep the lipid membrane in a fluid state. In most cases, buffer or salt solutions are used as aqueous media for efficient formation of a planar bilayer from vesicles, but pure water is also used in several previous studies [11,35,50,51]. Generally unilamellar vesicles with diameters of 30–200 nm are used for the vesicle fusion method. Giant unilamellar vesicles of 1–10 µm are also applied depending on the purpose [52,53,54,55] (for example, overlaying micropores on a substrate with a suspending lipid bilayer), but it is not suitable for the formation of a homogeneous SLB over the whole sample surface.

The interaction between lipid bilayer membranes and solid substrates is a quite influential factor during the vesicle fusion processes shown in Figure 1, as well as other factors, like the lipid components [56,57] and the size of vesicles [58,59,60,61,62], the solute concentration in the solution [40,58] and temperature. At the early stage of SLB researches, adsorption states of vesicles and transformation processes to a planar membrane was characterized *in situ* with label-free methods, such as quartz crystal microbalance [63], ellipsometry [64,65], surface plasmon resonance [64] and AFM [66]. There exist three typical processes for the vesicles adsorbed on substrate surfaces. One is that a single isolated vesicle, in some case after the fusion of a few vesicles, ruptures, resulting in a small SLB patch (Figure 1a) [58]. The SLB patches fuse with each other and/or with other vesicles and, finally, cover the whole surface. Another is that the surface is first covered with adsorbed vesicles until threshold coverage, and the rupture of a vesicle triggers the chain reaction of the vesicle spreading and the stimulation of the neighboring vesicles (Figure 1b) [67]. It is interesting that opposite dependences of the SLB formation efficiency on the size of vesicles were reported between the first and second processes. In the first process (Figure 1a), adsorbed vesicles larger than a critical diameter (~150 nm) transform a SLB disk [58]. In the second process (Figure 1b), the spreading of a SLB patch in adsorbed vesicles, smaller vesicles have higher efficiency of the SLB formation [59,60]. The third process of the adsorbed vesicles on substrates is an adhesive vesicular layer that stably forms, and the transformation to a planar membrane does not proceed (Figure 1c) [59,63]. Once the vesicular layer forms, it rarely turns to a planar membrane spontaneously, but there are several known ways to stimulate adhesive vesicles to rupture: addition of a reagent working as a membrane fuser (Ca^2+^ [58], poly-ethylene glycol [68]) or an amphipathic viral peptide [69], osmotic pressure [60], mild sonication [70] and freeze-and-thaw [71].

It is generally known that only a single layer of lipid bilayer membrane is formed by the vesicle fusion method using extruded or sonicated vesicles, unless specific attraction, e.g., avidin-biotin binding, DNA hybridization or covalent bond formation, is introduced between the first and second lipid bilayers [72,73]. Exceptionally, double SLBs are formed if the mica substrate is incubated in the vesicle suspension of dipalmitoylphosphatidylcholine (DPPC) or dimyristoylphosphatidylcholine (DMPC) at ~5 °C below the main phase transition temperature to L_α_, which is between the pretransition temperature (from gel to ripple phase) and the main phase transition temperature (from ripple phase to L_α_) [74,75]. The double layers and multilayers of lipid bilayer membrane are thermally stable once they are formed at solid-liquid interfaces [44,46,56,76,77,78,79,80]. The SLB formation by the vesicle fusion is an irreversible and dynamic process largely containing a kinetic aspect. Stable adsorption and deformation of vesicles, which are necessary processes for the transformation of vesicles to a planar membrane [58,81], proceed rather easily on solid surfaces, but not on the planar lipid bilayer already existing on the solid surfaces, because such strong and direct interaction results in the fusion of vesicles into SLB.

### 2.2. Effects of Substrates during the SLB Formation by the Vesicle Fusion Method

The transformation processes from vesicles to a SLB are strongly affected by the physical and chemical properties of the substrate surfaces, such as materials [40,59,60,63,67,82,83,84,85,86], chemical termination [25,26,62,63,87,88,89] and surface charges [54,90,91]. As a comprehensive parameter including several surface properties, the hydrophilicity of surfaces is useful to express the behavior of the vesicles on the surfaces. A hydrophilic surface is a prerequisite for the SLB formation from vesicles; thus, mica, glass, the oxidized layer on a Si wafer (SiO_2_/Si), quartz and TiO_2_ are generally used. Here, I describe an example how the degree of hydrophilicity affects the SLB formation by the vesicle fusion method [25].

**Figure 2:**
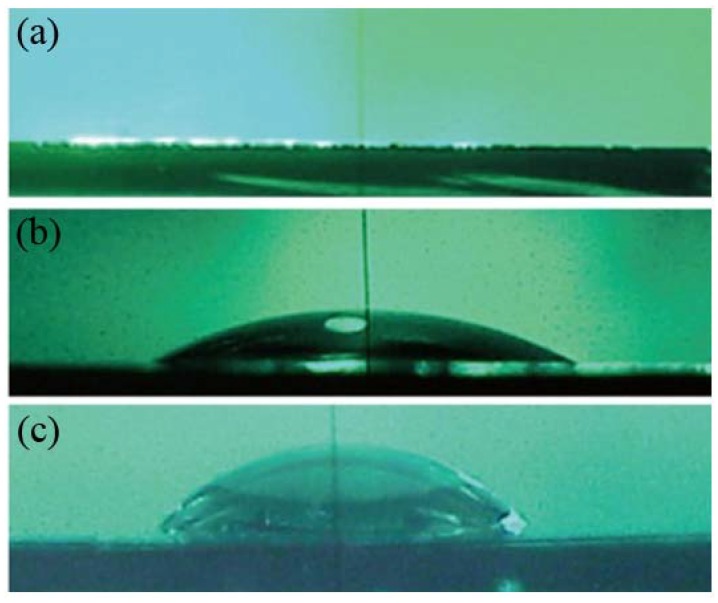
Water drops on chemically oxidized SiO_2_/Si surfaces (**a**) without annealing (WCA < 5°); (**b**) annealed at 700°C for 10 min (WCA = 24°); and (**c**) annealed at 700 °C for 60 min (WCA = 67°). Reproduced with modification from [25] by permission of the PCCP Owner Societies.

The surface of SiO_2_/Si and glass is hydrophilic, because hydroxyl groups (–OHs) exist on the surface. The density of –OHs is the critical factor determining the surface hydrophilicity if the surface morphology is the same; thus, the degree of surface hydrophilicity decreases with the reduction of –OHs density. Chemically oxidized SiO_2_ layer on Si(100) is highly hydrophilic with a water contact angle (WCA) below 5° (Figure 2a). The surface hydrophilicity decreases after thermal annealing under nitrogen because of the thermal desorption of –OHs as described below:

2OH(surface) → H_2_O(gas)↑ + O(surface) (1)

The degree of the hydrophilicity can be controlled with the annealing temperature and time (Figure 2b,c). It is because the irreversible desorption of –OHs occurs above 400 °C, and the amount of the irreversibly desorbed –OHs increases with the annealing temperature [92]. The surface roughness of the chemically oxidized SiO_2_/Si is R_a_ = 0.14 ± 0.02 nm, and little changes after the thermal annealing at least up to 700 °C.

**Figure 3:**
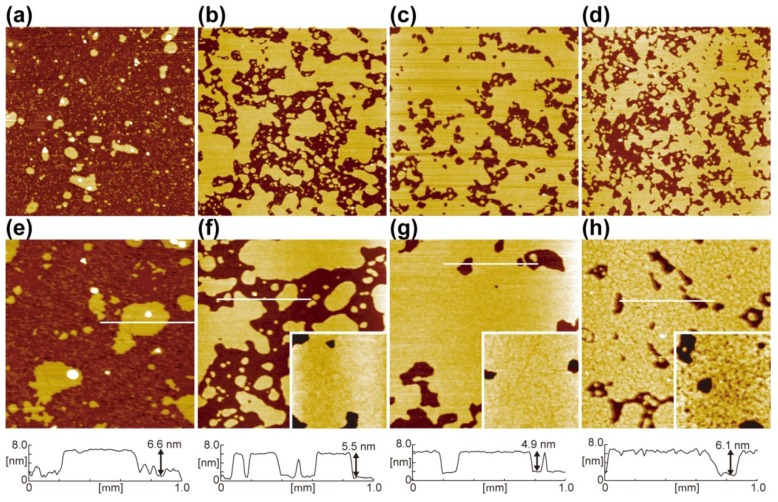
AFM topographies ((**a**)–(**d**): 5.0 × 5.0 μm^2^, (**e**)–(**h**): 2.0 × 2.0 μm^2^) and line profiles of hydrophilicity-controlled SiO_2_/Si surfaces after the incubation at 0.005 mg mL^−1^ of the 100-nm-filtred DMPC vesicle suspension. (**a**,**e**) Chemically oxidized SiO_2_/Si without annealing (WCA < 5°); (**b**,**f**) annealed at 480 °C for 10 min (WCA = 11°); (**c**,**g**) annealed at 700 °C for 10 min (WCA = 24°); and (**d**,**h**) annealed at 700 °C for 60 min (WCA = 67°); The insertions in (**f**–**h**) are magnified images (800 × 800 nm^2^) of the SLB surfaces. Reproduced from [25] with modification by permission of the PCCP Owner Societies.

Figure 3 shows hydrophilicity-controlled SiO_2_, prepared by the thermal annealing of SiO_2_/Si, as those in Figure 2, after incubation in a DMPC suspension under the same condition (100-nm-filtered vesicles, lipid concentration of 0.005 mg/mL, at 29 °C (higher than *T*_c_ of DMPC, 24 °C), for 60 min) in which a submonolayer of single DMPC-SLB with the height of 5–6 nm is obtained. The coverage of the SLB (*θ*_SLB_) is 0.12 (Figure 3a,e) on the SiO_2_/Si without annealing (WCA of the bare surface is <5° before the DMPC deposition as in Figure 2a). After the reduction of hydrophilicity, *θ*_SLB_ increases to 0.63 (Figure 3b,f) and 0.78 (Figure 3c,g) on the SiO_2_ surfaces pre-annealed at 480 °C for 10 min (WCA = 11°) and at 700 °C for 10 min (WCA = 24°), respectively. After further pre-annealing of the SiO_2_/Si at 700 °C for 60 min (WCA = 67°), the SLB forms efficiently (Figure 3d,h, *θ*_SLB_ = 0.67), but small pits appeared in the SLB (Figure 3h). The relation among the annealing condition, WCA of bare SiO_2_/Si, and *θ*_SLB_ after the incubation in the vesicle suspension is summarized in Table 1.

**Table 1:** Dependence of the efficiency of the SLB formation on the surface hydrophilicity controlled with the annealing conditions. All results from [25].

Annealing condition	Water contact angle	SLB coverage
Without annealing	<5°	0.12
480 °C for 10 min	11°	0.63
700 °C for 10 min	24°	0.78
700 °C for 60 min	67°	0.67

These results showed that the efficiency of the SLB formation increases with the reduction of the surface hydrophilicity. It is because of the reduction of hydration repulsion due to the desorption of –OHs from the SiO_2_ surface. The interaction energy between SLBs and solid substrates are dominated by the balance between the van der Waals attraction and hydration repulsion [93,94,95]. (In the case in Figure 3, electrostatic interaction is minor, because the zwitterionic head group of PC is close to neutral, and the slight surface potential is shielded at the salt concentration of ~160 mM.) Hydration repulsion is caused if the stable water layers around SiO_2_/Si surfaces and lipid head groups [96,97] overlap when a lipid vesicle approaches to the SiO_2_/Si surface. Thus, the water layer binding with surface –OHs via hydrogen bond works as the barrier for the approach and adsorption of lipid vesicles. Stable adsorption of vesicles is needed for the rupture of vesicles to transform to a SLB [58,59,60,67,81]; thus, the interruption of the vesicle adsorption process effectively affects the SLB formation kinetics. The removal of –OHs from SiO_2_/Si surfaces reduces the binding site for the hydrogen-bonding with water molecule; thus, weakening the hydration repulsion against vesicles, resulting in the efficient adsorption of vesicles and the SLB formation. Similar tendency in the acceleration of the SLB formation due to the desorption of surface –OHs is also reported on a glass substrate [89] and on silica particles [98].

I note that the hydrophilic substrate is a prerequisite condition for the SLB formation by the vesicle fusion method, but not always a sufficient condition. Cha *et al.* studied the SLB formation from the vesicle of phosphatidylcholine from egg yolk (eggPC) on alkanethiol self-assembled monolayers (SAMs) on gold with hydrophilic terminations (–NH_2_, –OH and –COOH) to control the surface charge density [90]. Fluid SLBs forms only when the density of charged termination (–NH_2_ or –COOH) is ≥ 80% mixed with the neutral termination (–OH). The surface of TiO_2_ is also hydrophilic, but previous studies from several groups showed that the adhesive vesicular layer of PC is formed predominantly on sputter-deposited TiO_2_ surfaces [40,59,60,69,71,82,83]. Rossetti *et al.* reported that a SLB is formed from vesicles on a sputter-deposited TiO_2_ when the vesicle contains 20% of negatively charged lipid, phosphatidylserine (PS), in the presence of Ca^2+^ ion in the buffer solution [40]. In this case, PS molecules preferentially distribute to the lower leaflet of SLB. On the other hand, Tero *et al.* used the single crystal TiO_2_(100) surface consisting atomic steps and flat terraces and found that a full-coverage SLB of PC is formed if the TiO_2_(100) surface is incubated in the sonicated vesicles (36 nm in diameter) at the lipid concentration higher than 0.025 mg/mL, while an adhesive vesicular layer is formed if the vesicle size is larger or the lipid concentration is lower [93,99]. The formation of the vesicular layer is irreversible, and once the vesicular layer forms, it does not transform to planar membrane, even if the surface with the vesicular layer is again incubated in the proper condition for the SLB formation [93]. Therefore, the suitable experimental condition for the SLB formation should be explored, depending on the substrate and lipid components. Recent reports show that the addition of an amphiphilic viral peptide [69] or freeze-and-thaw [71] stimulates the transformation of adhesive vesicles to a SLB on sputter-deposited TiO_2_ substrates. These methods may be also effective for the adhesive vesicles on the single crystal TiO_2_(100) surface.

In case the surface is covered with a hydrophobic alkyl chains (e.g., octadecyltrichlorosilane/SiO_2_, alkanethiol/Au and lipid monolayer/hydrophilic substrates), a vesicle zips-out at the interface between the two leaflets of lipid monolayer and transforms to a monolayer on the alkyl layer on the substrate surface [63,87,88,100,101]. Lenz *et al.* investigated the vesicle behavior on the surfaces of poly(dimethylsiloxane) (PDMS) with various hydrophilicity [102]. A hydrophobic PDMS surface gradually becomes hydrophilic, depending on the exposure time to oxygen plasma, and the states of eggPC vesicles on the PDMS surfaces vary from a monolayer on the hydrophobic surface, to no adsorption, to adhesive vesicles and, finally, to a bilayer on the most hydrophilic surface. In the context of the relation between the hydrophilicity of substrates and lipid bilayers, graphene oxide (GO) is an interesting material as a substrate for SLBs [103,104], because GO consists of nanometer scale patches of hydrophilic sp^3^ carbon domains and hydrophobic sp^2^ carbon domains [105,106]. The surface of GO is covered with adsorbed dioleoylphosphatidylcholine (DOPC) vesicles, but single or double SLBs form on GO in the presence of Ca^2+^ ion [103]. Interestingly, Furukawa *et al.* reported that the self-spreading eggPC-SLB avoids the GO flakes on SiO_2_/Si [107]. These are the extreme examples of the difference in the SLB formation between kinetically dominated (vesicle fusion) and thermally driven (self-spreading) processes.

## 3. Substrate Effects on the Molecular Diffusion in SLB

The fluidity of cell membranes is an important factor for biological reactions, many of which include the lateral molecular diffusion as a fundamental process for the lateral molecular transportation and formation and/or dissolution of two-dimensional domains [1,2]. The fluidity of SLBs is evaluated by fluorescence recovery after photobleaching (FRAP) [15,16], fluorescence correlation spectroscopy (FCS) [17] and single molecule tracking (SMT) [18]. Generally, diffusion in a macroscopic area on the order of several micrometers to 100 µm is evaluated with FRAP, while it is possible to detect the diffusion at a narrower region with FCS and SMT in principle. The lateral molecular diffusion in SLBs is always under the effect from substrates. It is reported from several groups that the diffusion coefficient (*D*) of lipids in SLB is two-to-three times smaller than that in free-standing lipid bilayers, such as giant vesicles [108,109]. It is to be noted that the experimentally obtained values of *D* vary according to the experimental method [109].

**Figure 4:**
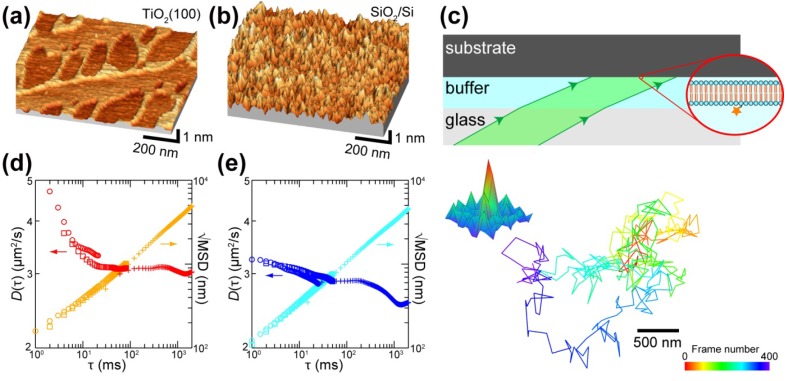
(**a**,**b**) AFM topographies of (**a**) step-and-terrace TiO_2_(100) and (**b**) amorphous SiO_2_/Si surfaces; (**c**) Schematic of the diagonal illumination for SMT on TiO_2_(100) and SiO_2_/Si, and the fluorescence intensity distributions and extracted 400 steps trajectories of a single Rb-DPPE molecule in DOPC-SLBs on TiO_2_(100) recorded at the time resolution of 996 µs (1004 fps); (**d**,**e**) Dependence of *D* and mean diffusion distance (√MSD) on time interval (τ) on (**d**) TiO_2_(100) and (**e**) SiO_2_/Si surfaces. Reprinted from [113] with modification with permission. Copyright 2011 American Chemical Society.

Artificial structures fabricated on solid substrates are used to control the lateral diffusion in SLBs [110,111,112]. Tero *et al.* investigated the lipid diffusion in DOPC-SLB on the TiO_2_(100) surface consisting of single atomic steps, which is the minimum structure one can fabricate, by SMT, and compared with that on SiO_2_/Si [113]. The TiO_2_(100) surface has linear single atomic steps (0.23 nm in height) and terraces, and oval pits also consisting of the single atomic step exist in the terraces (Figure 4a). The thermally oxidized layer on Si wafer is amorphous, and its surface has random protrusion with the peak-to-valley roughness of 0.6 nm (Figure 4b). The SLBs of DOPC, containing a 10^−9^–10^−8^ order of dipalmitoylphosphatidylethanolamine labeled with lissamine rhodamine B (Rb-DPPE), are formed on both substrates, and diffusion of each Rb-DPPE molecules is observed by SMT with the diagonal illumination setup (Figure 4c). In conventional SMT experiments, substrate materials are restricted to glass or quarts, because the excitation light is introduced from the backside of a substrate at the total internal reflection condition, and the fluorescence-tagged samples are illuminated by evanescent light [18,114]. The diagonal illumination setup with a sample substrate up-side-down shown in Figure 4c achieves the SMT measurement of SLB without the restriction on the substrate transparency and refractive index [113,115].

The single molecule image of Rb-DPPE is obtained, and its diffusion is tracked at the time resolution of 997 µs (1004 frames per second) at the maximum on the TiO_2_(100) surface (Figure 4c). It will be a powerful technique if one investigates the behavior of single molecules on a nanofabricated Si surface or a functional nanostructured oxide substrate. The SMT performed at the time resolution of 500 µs to 30 ms achieved lipid diffusion over the spatial and temporal ranges of 100 nm/ms to 1 µm/s (Figure 4d,e). The result on the step-and-terrace TiO_2_(100) (Figure 4d) clearly visualizes the crossover from the anomalous diffusion at the submicron region to the random diffusion at micrometer region and indicates the existence of ~200 nm compartments in the DOPC-SLB, which matches to the average inter-step distance of 248 nm on the TiO_2_(100). The SLB of PC on the step-and-terrace TiO_2_(100) precisely follows the atomic step structure of the substrate [93], because of the strong van der Waals attraction on TiO_2_ [93,94]. This membrane distortion above the substrate steps works as the barrier for the lateral diffusion of lipids in the SLB.

The value of *D* obtained at the time resolution of 2 ms on TiO_2_(100) is 4.7 µm^2^/s, which is larger than those evaluated at the time resolution of 30 ms (3.0 µm^2^/s and 2.5 µm^2^/s on TiO_2_(100) and SiO_2_/Si, respectively), is close to that reported in free standing membranes [108,109]. It means that the lipid diffusion is faster within the flat terrace than that across the atomic-scale membrane distortion induced by the substrate. Corrugation of the substrate surface, even on the subnanometer scale, possibly causes the impediments for the lipid diffusion SLB, and it may be the origin of the smaller diffusion coefficient in SLB than in free-standing bilayer membranes [108,109].

If one needs to exclude the effect of the substrate, a tethered lipid bilayer is the effective strategy [116,117], especially if proteins with large extra-membrane region are incorporated in the bilayer. A tethered bilayer is separated from the substrate by linker molecules, which tether the bilayer and the substrate. Generally used linker molecules are polymers covalently attached to the bilayer and the substrate, or avidin attached on the substrate binding with biotinyl lipids doped in the lipid bilayer. Dewa *et al.* extensively studied the diffusion properties of bacterial photosynthetic membrane proteins, light-harvesting complex 2 (LH2) and light-harvesting core complex (LH1-RC), in SLBs and tethered bilayers by FRAP [70,118,119]. The tethered bilayer of DOPC containing 1% biotinyl dioleoylphosphatidylethanolamine (N-biotinyl-DOPE) is prepared on an avidin-modified glass cover slip [120,121]. Proteoliposomes of LH1-RC and LH2 are prepared for the reconstruction of LH1-RC and LH2 into the SLB and tethered bilayer. The mobile fraction of LH1-RC, which has large extra-membrane region, is almost zero in SLB, but the mobile fraction of LH1-RC is improved to 40% in the tethered bilayer. Immobile proteins incorporated in lipid bilayers also work as the obstacles for the diffusion of lipids in the bilayer. Mobile fraction of Rb-DOPE is only 26% in the SLB containing LH1-RC. This means that immobile LH1-RC interrupts the lipid diffusion and also divides the SLB into disconnected patches. The mobile fraction of Rb-DOPE recovers to 100% in the tethered bilayer. The diffusion of LH2, which has small extra-membrane region, is less affected by the substrate, and its mobile fraction is 90% and 98% in the SLB and tethered bilayer, respectively. A fluid and continuous lipid bilayer system is achieved by using the tethered membrane. Meanwhile, if one is interested in the detailed structure of protein complexes, it is difficult to observe their structure using conventional AFM, because of the limitation of time resolution. However, high-resolution AFM images of each protein molecules can be obtained in SLB, where molecular diffusion is suppressed [122]. In this case, tethered bilayer and SLB provide complementary information, and thus, either should be chosen depending on the purposes.

## 4. Substrate Effects on the Domain Formation and Reactivity of SLB

Various kinds and sizes of domains and clusters exist in cell membranes and play key roles in signal transportation and molecular recognition through and on cell membranes [1,2]. A representative example is the concept of “raft domains”, but the relation between the properties of such domains and biological functions are still to be elucidated. SLBs will be also valuable as artificial reaction fields to study how two-dimensional assemblies of lipids and proteins affect biological functions. Phase separation and two-dimensional domain formation in artificial lipid bilayers have been extensively studied using artificial lipid bilayers [12,13,123,124,125] for the understanding of two-dimensional molecular organization in cell membranes from the viewpoints of physics and chemistry. In the case of SLB, pattering and fabrication techniques on solid substrates are applied to control the distribution of domains [126,127,128,129]. Yoon *et al.* fabricated a SiO_2_/Si surface with the arrays of nanosmooth regions in nanocorrugation with ~20 nm height and observed the phase separation of SLB consisting of DOPC, sphingomyelin (SM) and cholesterol (Chol) (1:1:1) on the patterned SiO_2_/Si surface [126]. Coalescence of the liquid ordered (L_o_) domains, which is rich in SM and Chol, selectively proceeds in the nanosmooth region, and the macroscopic L_o_ domains are confined in the smooth region. Parthasarathy *et al.* fabricated hog-backed structures with 2 µm in width an 50–200 nm in height, which induce a local curvature radius of 100 nm to the double SLBs on it. Cholesterol-rich L_o_ domains are excluded from the curved regions and aligned at the smooth regions [127]. In both cases, more fluid liquid-disordered domains than L_o_ domains preferentially distribute at the distorted region in the SLB, because of the corrugation of the substrate on the order of 10–100 nm. In some cases, however, much smaller structures of the substrates affect the morphology of two-dimensional domains. Next, I describe two examples in which the surface atomic structures affect the domains in SLB, especially relating to the interaction with peptides.

**Figure 5:**
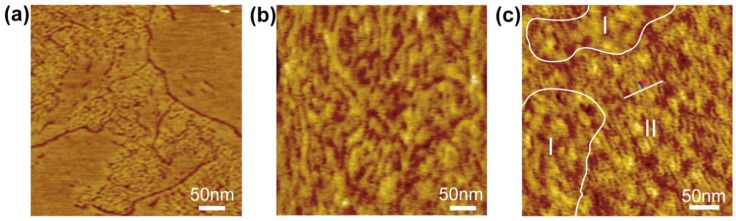
DPPC-SLB including gA (**a**) on mica, (**b**) on chemically oxidized SiO_2_/Si, and (**c**) tethered on avidin-modified SiO_2_/Si. The concentration of gA is 2 mol% in (**a**), (**b**), and 1 mol% in (**c**). Reprinted from [130] with modification with permission from Elsevier.

The first example is the two-dimensional domains of gramicidin A (gA) in DPPC-SLB reported by Lei *et al* [130]. Gramicidin A is a polypeptide consisting of fifteen amino-acid residues and widely used as a model ion channel [131,132]. It is known that gA two-dimensionally assembles in artificial lipid bilayers and monolayers depending on the concentration of gA and the component and preparation method of the lipid membranes [133,134,135]. Figure 5a shows the DPPC-SLB incorporating 2 mol% of gA on mica prepared by the vesicle fusion method. Aggregation of gA is observed as depletion because the length of the gA helix is smaller than the thickness of a DPPC monolayer in the gel phase [131,132,136]. Definitely, two types of depletion, dot-like and string-like ones, exist as reported in [134]. The domains, including the dot-like depletions, are separated from the depletion-free DPPC-SLB domains, and the string-like depletions exist at their boundaries. On a chemically oxidized SiO_2_/Si (roughness: R_a_ = 0.14 nm), on the other hand, the aggregation of gA randomly distributes in the DPPC-SLB (Figure 5b). Clear separation between the gA-free and gA-incorporated regions on mica is not observed on the SiO_2_/Si surface, and the size and shape of the depletion are irregular. The gA aggregates show different morphology in a tethered lipid bilayer (Figure 5c). In the tethered bilayer of DPPC + N-biotinyl-DOPE + gA (99:1:1) on avidin-modified SiO_2_/Si [120,121], there is a clear separation between the gA-free DPPC-SLB region (I in Figure 5c) and the gA-incorporated region, including depletions (II in Figure 5c). Only the dot-like depletion is observed in the region II, and the string-like depletion, which appears on mica (Figure 5a), is not observed at the boundary between the regions I and II. The morphology of the region I is similar to that of the gA-free DPPC-SLB on SiO_2_/Si. The depths of these depressions attributed to gA in the three samples in Figure 5 are similar (0.25 ± 0.09 nm on mica, 0.38 ± 0.17 nm on SiO_2_ and 0.34 ± 0.15 nm in tethered bilayer) and seem independent on the substrate. These results show that the assembly of gA similarly proceeds on molecular level (hexamer is proposed as the minimal unit [133,134,135]), but their assemblies on the mesoscopic scale are strongly affected by the substrate surface.

**Figure 6:**
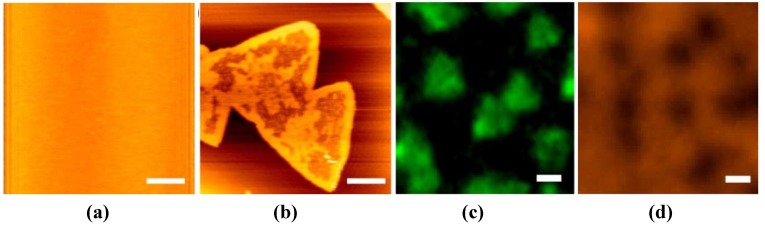
(**a**,**b**) AFM topographies of SM + Chol + GM1-SLBs on mica (**a**) just after preparation and (**b**) after incubation at 37 °C for 24 h; (**c**,**d**) Fluorescence images of SM+Chol+GM1-SLBs similarly prepared to Figure 6b assaying (**c**) SM by doping NBD-SM and (**d**) GM1 by addition of CTX-B labeled with Alexa555. Scale bars are 1 µm. Reprinted from [137] with modification with permission from Elsevier.

The second example is the domain formation of SLBs containing a glycolipid, ganglioside GM1 (GM1) and their reactivity to the polymerization of amyloid β peptides (Aβ). GM1 forms a seed complex with Aβ and accelerates the formation of toxic Aβ oligomers and fibrils, which are implicated as a cause of neuronal death in Alzheimer’s disease [137,138]; thus, SLBs containing GM1 are expected as a model reaction field to understand the fundamental processes of Aβ oligomerization and fibril formation. Mao *et al.* investigated the surface morphology and domain formation of the SLB of SM + Chol + GM1 (40:40:20) on the chemically oxidized SiO_2_/Si and mica surfaces [139,140]. The ratio of the lipids is that active for the growth of the Aβ fibril in the liposome assay reported by Yanagisawa *et al.* [138,139]. The SM + Chol + GM1-SLB on mica is flat and uniform just after the preparation (Figure 6a) as well as on SiO_2_/Si, but unique triangular domains appear in the SM + Chol + GM1-SLB only on the mica substrate (Figure 6b) after incubation at 37 °C for 24 h [139]. The assays using dye-labeled SM (NBD-SM) and cholera toxin B (CTX-B), which strongly associates with GM1 [141,142], showed that SM and GM1 preferentially distributed in and out of the triangular domains, respectively (Figure 6c,d). The result of FRAP shows that both the triangular domains and the outer regions are fluid; thus, the triangular domain contains mainly SM and Chol, while the outer regions mainly consist of Chol and GM1. In the SM + Chol + GM1-SLB prepared on SiO_2_/Si in the same condition as that shown in Figure 6b–d, NBD-SM and CTX-B randomly and uniformly distribute in the SLB. Therefore, the formation of the triangular domain in SM + Chol + GM1-SLB on mica is a substrate-induced phenomenon. The surface of mica, which has six-fold symmetry, causes the domain formation and works as the template of the domain structure, because all triangles align in the same direction.

**Figure 7:**
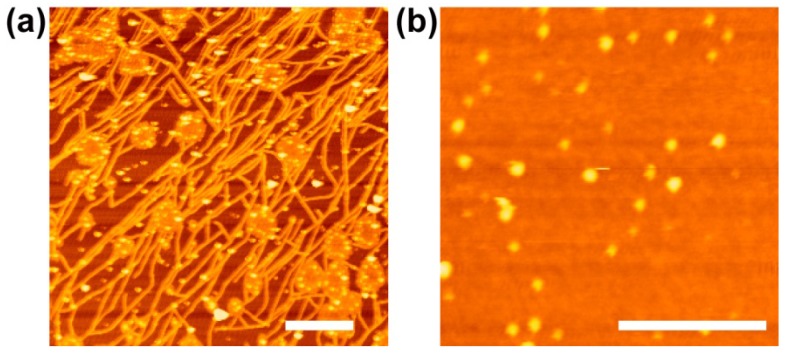
AFM topographies of SM + Chol + GM1-SLBs on (**a**) mica and (**b**) SiO_2_/Si after incubation in the presence of Aβ of 2 µM at 37 °C for 24 h. Scale bars are 1 µm. Reprinted from [137] with modification with permission from Elsevier.

It is interesting to point out that the chemical activity to the polymerization of Aβ peptide on the SM + Chol + GM1-SLB is also strongly affected by the substrates (Figure 7) [138]. GM1 forms a seed complex with Aβ and accelerates the formation of Aβ oligomers and fibrils, as mentioned above [137,138]. SM + Chol + GM1-SLBs on mica and SiO_2_/Si are incubated in the presence of Aβ of 2 µM at 37 °C for 24 h. The fibrils of Aβ grow on the SLB on mica (Figure 7a), even though the concentration of Aβ is 25-times lower than that reported in the liposome assay at the same lipid composition (50 µM, [137,138]). On the SLB on SiO_2_/Si, particle-like aggregations are observed (Figure 7b), but the growth of the fibril does not proceed independently of the concentration of Aβ. The results show that the functions of SLBs as a biochemical reaction field also depends on the surface properties of the substrates under the SLB. The mechanism of the difference in the activity to Aβ fibril growth between the SLBs on mica and SiO_2_/Si is still to be elucidated, and detailed studies about the molecular conformation and clustering state of GM1 will be needed [143].

## 5. Outlook

Supported lipid bilayers are under the influence of solid substrates, though the water layer between the SLBs and substrates retains the fluidity and dynamics in SLBs. It provides the advantage that various surface fabrication techniques (e.g., photolithography, microcontact printing, focused ion beam, *etc.*) are available to manipulate and control SLBs and their properties, but always care should be taken for the denaturing of biological molecules and artificial phenomena. The hydrophilicity of the substrate will be one of the critical factors determining the SLB-substrate interaction, because it affects the stability and the thickness of the water layer between the SLBs and substrates and, also, because a lipid bilayer itself is a self-assembled structure due to the hydrophilic-hydrophobic interaction surrounded by the water molecules bound to the head groups with hydrogen bonds. The hydration repulsion on hydrophilic substrates is dominated by the density of surface –OH groups and can be controlled to some extent by a simple annealing procedure. Recent developments of AFM achieved the direct visualization of the three-dimensional distribution of water layers above solid substrates and lipid bilayers [144,145], and this information will be essential to understand the water-mediated interaction between SLBs and substrates.

I also summarized several examples of the effects of the structures and materials on the lateral molecular diffusion and two-dimensional domain formation in SLBs. I emphasize that all of the substrate materials, SiO_2_/Si, mica and TiO_2_ are biologically inert and atomically flat, but cause significant differences to the phenomena in SLB. The SMT measurement with high spatiotemporal resolution indicates that the corrugation on the substrate surface on the atomic scale possibly causes the interruption of the lateral diffusion in SLBs and that it may be the origin of the difference in *D* between SLBs and free-standing bilayers. It will be one of the origins of the substrate-dependent domain formation, because the molecular diffusion is a fundamental step influential to the nucleation and growth processes of domains. In the case of gA aggregation in DPPC, microscopic structures of gA seem independent of substrates, but their assemblies on the order of 10–100 nm are drastically dependent on substrates. The triangular domains formed in SM + Chol + GM1−SLB are induced and templated by the mica surface. In this case, activity to the Aβ fibril growth indicates that the molecular conformation and/or clustering state of GM1 are also strongly affected by the substrates. It is assumed that the “raft” domains are rich in SM, Chol and GM1, and the importance of short-lived microdomains and clusters is emphasized in the concept of the raft [1,2,146]. Tuning of biochemical activities of SLB applying the functions of substrates will provides artificial reaction fields to investigate the details of the active species in bilayer membranes. SMT with high-time resolution will play a role in characterizing the clustering states of lipids and proteins in SLBs, as well as AFM.
